# Surrogate markers in antiangiogenesis clinical trials

**DOI:** 10.1038/sj.bjc.6601035

**Published:** 2003-07-01

**Authors:** D W Davis, D J McConkey, J L Abbruzzese, R S Herbst

**Affiliations:** 1 Department of Cancer Biology, Unit 173, The University of Texas MD Anderson Cancer Center, 1515 Holcombe Boulevard, Houston, TX 77030, USA; 2 Department of Gastrointestinal Medical Oncology, Unit 426, The University of Texas MD Anderson Cancer Center, 1515 Holcombe Boulevard, Houston, TX 77030, USA; 3 Department of Thoracic/Head & Neck Medical Oncology, Unit 432, The University of Texas MD Anderson Cancer Center, 1515 Holcombe Boulevard, Houston, TX 77030, USA

**Keywords:** laser scanning cytometry, biopsy, clinical trial, biomarkers, PET

## Abstract

Novel antiangiogenic agents currently being developed may ultimately be more effective against solid tumours and less toxic than cytotoxic chemotherapy. As a result of the early clinical trials of angiogenesis inhibitors, investigators are beginning to appreciate the complexity of targeting angiogenesis and the realisation that developing clinically useful antiangiogenic therapy will be more challenging than originally thought. It is now apparent that new methods and surrogate markers to assess these agents' biological activity are crucial for their successful development. This review summarises the currently available clinical data on the development of surrogate markers of angiogenesis inhibitors.

Tumour angiogenesis is a complex process modulated by positive and negative soluble factors released by tumour and host cells and is essential for neoplastic growth and metastasis (Fidler and Ellis, 1994; Folkman, 1995). Many angiogenic factors, including vascular endothelial growth factor (VEGF), platelet-derived endothelial cell growth factor (PDGF), basic fibroblast growth factor (bFGF), and interleukin-8, have been shown to promote endothelial cell survival, proliferation, and differentiation. These proangiogenic factors are essential for capillary morphogenesis during angiogenesis (Hanahan and Folkman, 1996; Benjamin and Keshet, 1997). The microvascular endothelial cell has become an important target in cancer therapy because tumours recruit endothelial cells early during angiogenesis (Witte *et al*, 1998). In addition, endothelial cells do not develop drug resistance (Boehm *et al*, 1997; Kerbel, 1997) and are easily accessible through the circulation. Furthermore, an agent that collapses a single capillary may amplify an antiangiogenic effect within the tumour (Inoue *et al*, 2000; Ozawa *et al*, 2001). These potentially therapeutic benefits of antiangiogenesis therapy have led to the development of over 200 anticancer agents that are in various stages of clinical investigation (at least 45 agents are in clinical trials for the treatment of solid tumours; www.nci.nih.gov/search/clinica ltrials).

Preclinical studies suggested that angiogenesis inhibitors are cytostatic (growth-delaying) rather than cytotoxic. Traditionally, the maximum tolerated dose of a cytotoxic agent (determined by its dose-limiting toxicity) provided an estimation of the active dose range for subsequent clinical studies (Simon *et al*, 1997). However, for antiangiogenic agents it is uncertain whether doses associated with clinical toxicity correlate with an antiangiogenic effect. Thus, because for antiangiogenic agents there is an uncertain relation between toxicity and response, determining the optimal biological dose (OBD) of angiogenesis inhibitors depends on a different logic than conventional cytotoxic chemotherapy. Several clinical studies have shown that angiogenesis inhibitors are optimal at doses well below the maximum dose studied (Brewer *et al*, 2000; Rowinsky *et al*, 2000). Clinical investigation of angiogenesis inhibitors has accentuated the need to develop new biological markers to evaluate novel agents with putative antiangiogenesis activity.

The complexity of the biology underlying tumour angiogenesis has generated various approaches to measuring an antiangiogenic effect (Figure 1

**Figure 1:**
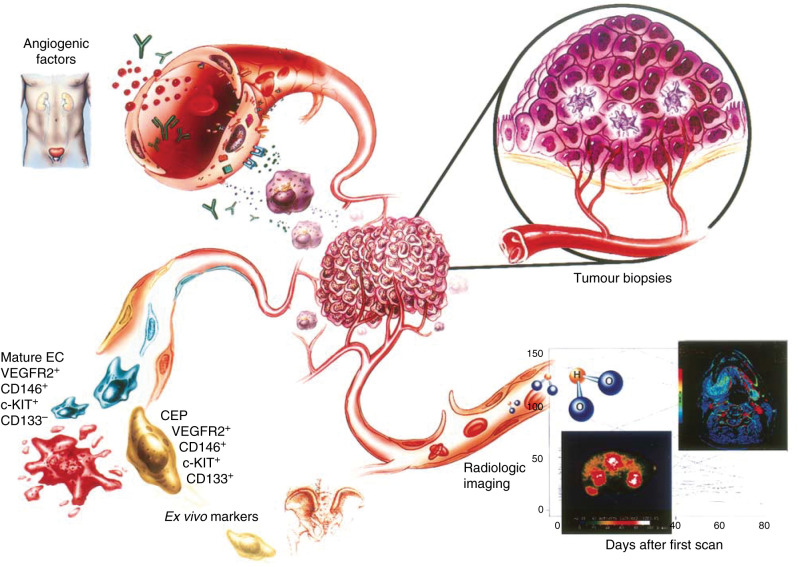
Novel assays for measuring surrogate markers of tumour angiogenesis activity. The complex biology of angiogenesis inhibitors has accentuated the need for developing technologies that can be used to assess the effects of biological markers. A compilation of data from multiple assays including measuring angiogenic factors in serum, plasma, and urine; tumour biopsy analysis; radiologic imaging; and, recently, *ex vivo* analyses of isolated peripheral blood cells (labelled circulating endothelial cells) may facilitate defining the OBD for subsequent clinical studies of angiogenesis inhibitors.

). The most commonly used surrogate assays measure specific serum, plasma, and/or urine levels of angiogenic factors that are hypothesised to be direct or indirect targets of the antiangiogenesis agent. Emerging radiologic techniques include positron emission tomography (PET), magnetic resonance imaging, dynamic computed tomography, and three-dimensional ultrasound. Each of these techniques is being used to assess changes in tumour blood flow, vascular permeability and in some cases metabolism (measured by PET). Other creative surrogate assays include *ex vivo* analyses of circulating endothelial precursor cells (CEPs) that are recruited from the bone marrow during tumour neovascularisation (Lyden *et al*, 2001; Reyes *et al*, 2002). Perhaps the most direct approach for determining the biological activity of antiangiogenic therapy involves the analysis of tumour biopsies before and at specified time points after the administration of the antiangiogenic agent. Quantifying drug–target interactions at the cellular level (e.g. measuring phosphorylation of VEGFR-2 following administration of a VEGFR-2 tyrosine kinase inhibitor) are crucial to determine that the target inhibition has been achieved for a given angiogenesis inhibitor. Most importantly, an analysis of tumour biopsies permits the direct measurement of apoptosis in endothelial cells (an important end point of antiangiogenic therapy) and the consequences of endothelial cell death on surrounding tumour cells. Although these assays are still being developed and the surrogate markers summarised in this review must be scrupulously validated, we believe that a combination of such assays can be used to define the OBDs of angiogenesis inhibitors. Advantages and disadvantages of these methods are summarised in Table 1

**Table 1:** Comparison of methods used to monitor surrogate markers of antiangiogenic activity

**Method**	**Advantages**	**Disadvantages**
Angiogenic factors	Noninvasive (Urine/serum/plasma)	Effects following inhibition of receptor unknown
	Quantitative	Interpatient variability, tumour specific
PET, MRI, CT, US	Assessment of tumour size, blood flow and volume	Expensive, not routinely available
	Determine disease-specific response	Effects on tumour blood flow not validated
Biopsy analysis	Provides proof of concept	Invasive
	Quantitative (LSC-based)	Effects of tumour heterogeneity unknown
*Ex vivo* markers	Noninvasive, feasible	Difficult to detect rare cell populations
	Quantitative	Requires immediate analysis

.

## SERUM, PLASMA, AND URINE FACTORS

Proteins thought to be important mediators of angiogenesis can be measured in serum, plasma, and urine by using enzyme-linked immunosorbent assays. A significant change in the level of angiogenic protein(s) after initiation of treatment might provide an early indication of antiangiogenic activity before clinically demonstrable reductions in tumour size. Elevated levels of angiogenic growth factors, proteases, and endothelial adhesion molecules have been detected in sera of patients with malignant disease (Dirix *et al*, 1997). These important promoters of tumour angiogenesis include VEGF (Takahashi *et al*, 1994), bFGF (Fujimoto *et al*, 1995), urokinase-type plasminogen activator and its soluble receptor (Xu *et al*, 1997), E-selectin and vascular cell adhesion molecule-1 (VCAM-1) (Banks *et al*, 1993), and von Willebrand's factor (vWF) (Gadducci *et al*, 1994). Of the many mediators of angiogenesis, VEGF and bFGF are thought to play the most important roles and thus have been frequently measured as potential surrogate markers of antiangiogenic activity in many Phase I/II studies.

In a Phase I dose-escalation study of SU5416, a small-molecule inhibitor of the VEGF receptor-2 tyrosine kinase, urinary VEGF and bFGF levels were found to vary 1–2-fold during the course of therapy but did not correlate with response or treatment dose (Stopeck *et al*, 2002). Interestingly, the baseline urine VEGF levels were significantly lower in the four patients that clinically responded than in the 18 nonresponders. However, bFGF levels were not significantly affected. The effects of angiogenesis inhibitors on the level of angiogenic factors may be drug- or tumour-type specific and may be dependent on the level of proteins detectable in different bodily fluids. For example, urine, serum, and plasma levels of bFGF and VEGF were measured in patients with renal cell carcinoma enrolled in a Phase II study of razoxane (Braybrooke *et al*, 2000), an antiangiogenic topoisomerase II inhibitor derived from the chelating agent EDTA. The levels of urinary VEGF significantly increased in patients who developed progressive disease but not in those with stable disease. However, no significant increases were observed in serum VEGF after one cycle of therapy, although baseline levels of serum VEGF were significantly higher in patients who subsequently developed progressive disease than in those with stable disease. Other mediators of angiogenesis (including serum VCAM-1, vWF, and urokinase plasminogen activator soluble receptor) hypothesised to be indirect targets of razoxane were significantly higher in progressive disease patients than in stable disease patients before and after one cycle of treatment (Braybrooke *et al*, 2000). In a Phase II study of the antiangiogenic agent thalidomide, 16 of 36 patients with recurrent, high-grade gliomas that had radiographic and clinically stable disease had either stable or decreased serum bFGF levels (Fine *et al*, 2000). Interestingly, serum bFGF levels significantly correlated to survival; patients whose serum bFGF significantly decreased (compared to baseline) survived approximately twice as long (43 weeks) than in those patients whose levels increased beyond the third week of therapy. In our Phase I study of endostatin, two of 25 patients experienced minor anticancer effects, but their serum VEGF, bFGF, VCAM-1, and E-selectin levels did not significantly change after treatment (Herbst *et al*, 2002a).

An important family of proteinases essential for the degradation of the extracellular matrix (ECM) and the basement membrane during angiogenesis include the family of matrix metalloproteinases (MMPs) (Liotta and Stetler-Stevenson, 1990). Proteolytic activation of the MMPs is regulated by the tissue inhibitor metalloproteinase (TIMP) family of proteins (Gomez *et al*, 1997). Matrix metalloproteinases also increase the bioavailability of factors, such as bFGF that are essential for the ECM (Haro *et al*, 2000). In patients, high levels of MMP expression have been shown to correlate directly with metastasis (Liotta and Stetler-Stevenson, 1990; Brown *et al*, 1993) and poor prognosis (Murray *et al*, 1996; Vihinen and Kahari, 2002). Therefore, inhibition of MMPs has become an important target for antiangiogenesis therapy, and the proteins themselves have become surrogate markers of antiangiogenic activity.

Patients enrolled in a Phase I study of MM1270, a nonspecific inhibitor of MMPs, had a significant increase in serum levels of MMP-2, MMP-9, TIMP-1, TIMP-2, and bFGF after one cycle of treatment (Levitt *et al*, 2001). Importantly, stable disease was observed in 19 of 92 patients. Other indirect surrogate markers that were measured but failed to demonstrate any significant change after treatment included VEGF, VCAM-1, soluble urokinase plasminogen activator receptor, and cathepsin B and H (proteases involved in the degradation of the ECM). In another Phase I study, of BAY12-9566, an MMP inhibitor that targets MMP-2, -3, and -9, only changes in the plasma levels of TIMP-2 were significantly related to dose (Rowinsky *et al*, 2000). Ironically, despite achieving biologically relevant plasma concentrations of BAY12-9566, changes in the levels of MMP-2 and MMP-9 were not significantly affected by the dose. It is possible that negative feedback loops become activated, such that increasing inhibition of the enzyme results in further production. However, in a Phase I study with COL-3, changes in plasma MMP-2 were significantly related to dose when compared to patients with progressive disease *vs* those with stable disease (Rudek *et al*, 2001). The direct effect on MMP-2 is not surprising because COL-3, which was derived from tetracycline, is a competitive inhibitor of MMP-2 (Seftor *et al*, 1998). Serum levels of VEGF and bFGF were also measured but failed to demonstrate any significant correlations.

Other surrogate serum markers include specific target-related proteins associated with the mechanism(s) of action of the angiogenesis inhibitor or the disease under study. In a Phase I study of tetrathiomolybdate (Brewer *et al*, 2000), an anticopper agent developed for Wilson's disease, serum ceruloplasmin was used as a surrogate marker to monitor total body copper. The goal of the study was to reduce ceruloplasmin to 20% of baseline value. Five of six patients with stable disease reached the target range. In another study, of TNP-470, an analogue of the antibiotic fumagillin, prostate-specific antigen (PSA) was measured to monitor antitumour activity in patients with progressive androgen-independent prostate cancer (Logothetis *et al*, 2001). Surprisingly, a reproducible transient increase in PSA concentrations was observed in some patients treated and rechallenged with TNP-470; however, the biological effect on PSA did not appear to be influenced by dose. Urine bFGF levels measured sequentially revealed no significant relation to dose or baseline serum bFGF concentrations.

Thus far, only a few angiogenic factors evaluated in clinical studies have proven useful for prognosis rather than monitoring response (Table 2

**Table 2:** Summary of changes in angiogenic factors compared with patient outcome

**Agent**	**Pat**^a^	**Outcome**	**Fluid**^b^	**VEGF**	**bFGF**	**VCAM-1**	**v-WF**	**Thm**^c^	**Esel**	**uPAsr**	**MMP-2**	**MMP8**	**MMP9**	**TIMP1**	**TIMP2**
Razoxane	35	StD(11)	U, P, S (56)	S, Pre PD>StD^d^	S, Post PD>StD^d^	S, Post PD>StD^d^	S, Pre PD>StD^d^			P, Pre PD>StD^d^					
				U, PD Pre vs Post^e^	U, Pre PD>StD^d^	S, PD Pre vs Post^e^	S, Post PD>StD^d^			P, Post PD>StD^d^					
MM1270	75	StD(19)	P(28)	Nc	w/AUC^e^	Nc					w/AUC^e^	Nc	W/*C*_max_^e^	W/AUC^e^	W/AUC^e^
BAY 12-9566	21	No response	P(29)								Nc		Nc		Post>Pre
COL-3	35	StD(8)	S, P(28)	S, Nc	S, Nc						P, Post PD>StD		P, Nc		
Endostatin	25	StD(1), MR(1)	S(28,56)	Nc	Nc	Nc			Nc						
TNP-470	12	No response	U, S(28)		Nc			S, Nc	Nc						
SU5416	22	StD(3), MR(1)	U(36)	Pre>Post^e^	Nc										
Thalidomide	16	PR(2), MR(2), StD(12)	S, U(56)	Nc	S, Decrease^d^, U, Nc										

a Patients studied.

b P=Plasma, S=Serum, U=Urine (days post-treatment).

c Thrombomodulin.

d Significant differences.

e Significant increase. Nc=no significant change; Pre=before treatment; Post=after treatment; PD=progressive disease; StD=stable disease; MR=minor response; PR=partial response; AUC=area under concentration curve.

). Validation of factors from homogeneous types of cancer may facilitate identifying new surrogate markers. Further studies are needed to refine methods and develop specific surrogate factors that may be more sensitive to the effects of angiogenesis inhibitors.

## LASER SCANNING CYTOMETRY OF TUMOUR BIOPSIES

**Figure 2:**
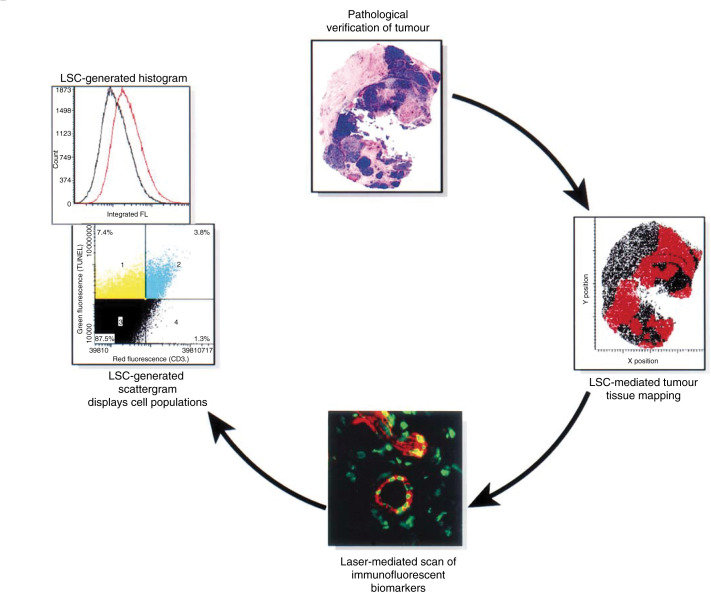
Quantitative analysis of surrogate markers in tumour biopsies using LSC. Pathological verification of biopsy samples is essential for mapping tumour regions and excluding normal cells from the analysis. Automated lasers detect individual cells within the mapped region of interest based on multicolour immunofluorescence staining of biomarkers. Each cell is plotted on a scattergram based on its relative fluorescence intensity. Laser scanning cytometry-generated scattergrams display the percentage of cell populations, for example, apoptotic endothelial cells. Alternatively, cellular protein expression levels, for example, phosphorylated VEGF receptor-2, may be measured by histogram analysis. (Immunofluorescent image appears with permission of Eaton Publishing, Westborough, MA 01581, USA; Cover, BioTechniques Vol. 28, No. 6 (June 2000).)

One important approach for evaluating antiangiogenesis activity is monitoring surrogate markers directly in tumour biopsy specimens. The ability to measure molecular changes (such as phosphorylation of receptor tyrosine kinases associated with drug–target interactions before and after therapy) can provide early proof of whether the biologic agent has successfully reached its hypothesised target. Some of the most compelling studies involved quantitative analysis by laser scanning cytometry (LSC) (Figure 2

). Laser scanning cytometry is analogous to fluorescence-activated cell sorting in that LSC can scan an entire tumour cross-section, simultaneously interrogating each cell to provide quantitative information such as the percentage of tumour-associated apoptotic endothelial cells or levels of cellular protein expression.

Laser scanning cytometry-mediated analysis of the effects of SU5416 and SU6668 in biopsy specimens obtained from Phase I/II studies demonstrated that these small molecules did not significantly inhibit phosphorylation of their primary target, VEGF receptor-2, or significantly increase apoptosis in tumour-associated endothelial cells (Davis *et al*, in preparation). In contrast, preclinical studies suggested that these small molecules had great potential, as they induced significant levels of apoptosis in endothelial cells, consequently inhibiting tumour growth (Shaheen *et al*, 1999). These data may explain the lack of clinical activity observed in the clinical studies (SU5416 Phase II at University of Chicago and SU6668 Phase I at MD Anderson Cancer Center), but most importantly, the results demonstrate that the validity of VEGF receptor as a therapeutic target has not yet been adequately tested.

In a Phase I dose-escalation study of endostatin, LSC-mediated analysis was used to quantify changes in markers of antiangiogenic activity 56 days after treatment (Herbst *et al*, 2002b). Changes in endothelial cell death, microvessel density, and tumour blood flow, measured by PET (see Radiologic imaging), were all statistically significant at intermediate doses of the drug (approximately 250 mgm^2^d) (Davis *et al*, submitted). In addition, LSC-mediated analysis of additional surrogate markers hypothesised to be effected by endostatin including nuclear hypoxia-inducible factor-1*α* and endothelial cell Bcl-2 levels peaked at similar doses of endostatin but failed to reach statistical significance. Importantly, two patients treated at intermediate doses of endostatin had minor antitumour responses (Herbst *et al*, 2002a).

The advantages of LSC-based quantification over standard manual methods include its automation, its higher sensitivity, and ability to analyse tens of thousands of cells rather than the hundreds of cells in a few high-powered microscopic fields. For example, in another Phase I study of endostatin involving tumour biopsy specimens, Mundhenke *et al* (2001) detected apoptosis in tumour-associated endothelial cells but were not able to discern clear patterns of cell death and vascular changes, perhaps because of low analytical power. In addition, surrogate markers in skin biopsy samples were measured to determine the feasibility of monitoring antiangiogenic activity. Unfortunately, these markers failed to demonstrate any significant correlation with endostatin dose. However, this study did reveal that analysis of skin biopsies might be useful for monitoring potential toxicity of angiogenesis inhibitors (Mundhenke *et al*, 2001). Indeed, the overall lack of toxicity observed in patients treated with endostatin was consistent with the analyses of endothelial cell biomarkers in wound sites.

## RADIOLOGIC IMAGING

Routine radiographic imaging techniques (magnetic resonance imaging, dynamic computed tomography, and three-dimensional ultrasound) are useful for conventional measurement of disease and evaluating the effect of a biologic agent on tumour size (Braybrooke *et al*, 2000; Rudek *et al*, 2001; Stopeck *et al*, 2002). However, changes in tumour size may occur long after initiation of therapy or in some cases not at all. In addition, the effects of antiangiogenesis inhibitors on disease stabilisation may be attributable to continuous, low doses of the drug. Therefore, developing surrogate markers by employing noninvasive imaging would facilitate defining the OBD, especially if the antiangiogenic effects were optimal at intermediate (low) doses or in specific cancers. For example, three-dimensional ultrasound depicting vascularity revealed a 4.4-fold decrease in tumour blood flow in a rib metastasis from renal cell carcinoma after 8 weeks of tetrathiomolybdate therapy, and dynamic computed tomography scan imaging confirmed that the lesions with decreased blood flow were stable (Brewer *et al*, 2000). In a Phase I study of endostatin, PET imaging was used to monitor changes in tumour blood flow [^15^O](H_2_O) and tumour metabolism [^18^F](FDG-fluorodeoxyglucose) 28 and 56 days after endostatin therapy (Herbst *et al*, 2002b). It was hypothesised that the antiangiogenic effect of endostatin would decrease tumour blood flow and metabolism. Interestingly, the study did reveal that endostatin decreased tumour blood flow having maximal effects between 180 and 300 mg m^2^d. However, tumour metabolism appeared to have a complex relation with tumour blood flow, increasing at a dose of approximately 180 mgm^2^d before decreasing at doses >300 mgm^2^d. Analysis of the tumour blood flow data using a quadratic polynomial model showed that endostatin-induced changes in tumour blood flow were significantly related to dose at 56 days (Davis *et al*, submitted). Importantly, the 95% confidence interval identified for blood flow overlapped the OBD (250 mgm^2^d) determined from the tumour biopsy studies. Thus, integration of surrogate marker data along with imaging studies was crucial for assessing the OBD of recombinant human endostatin.

## *EX VIVO* MARKERS

The heterogeneity of tumour biology and drug delivery to solid tumours may lead to variability in the results, making the interpretation of tumour biopsy and imaging data extremely challenging. Consequently, other creative strategies are being developed to monitor surrogate markers of antiangiogenic activity, for example, *ex vivo* analyses of isolated peripheral blood cells. In one study, a cytokine release assay was used to measure the effect of MM1270 on release of tumour necrosis factor-*α* from *ex vivo*-stimulated peripheral blood cells (Levitt *et al*, 2001). Matrix metalloproteinase inhibitors have been shown to regulate TNF-*α* activity (Gearing *et al*, 1994), and hence, it was hypothesised that MM1270 treatment would inhibit TNF-*α* release from stimulated whole blood cultures. Although there was slight inhibition of TNF-*α* release during MM1270 treatment, the results were not statistically significant, and there was no relation to dose. Furthermore, attempts to demonstrate apoptosis in endothelial cells isolated from patients after exposure to angiogenesis inhibitors, for example, endostatin, failed (Herbst *et al*, 2002a). Unfortunately, *ex vivo* analyses such as these remain complex and their utility may depend on several factors including the inhibitor's relative specificity for tumour-associated endothelial cells, the sensitivity of the assay to detect effects on rare cell populations, and limited exposure time of the drug to have an effect on peripheral blood cells before they are isolated.

The use of flow cytometry to quantify activated circulating endothelial cells from the peripheral blood of cancer patients has provided another approach to assessing the effects of antiangiogenic activity (Mancuso *et al*, 2001). Recently, work has shown that VEGF-dependent mobilisation of bone marrow-derived CEPs can contribute to tumour neovascularisation (Lyden *et al*, 2001). Heymach *et al* and others hypothesised that angiogenesis inhibitors decrease the number of CEPs (Bertolini *et al*, 2001) and increase the number of mature circulating endothelial cells (CECs) by causing shedding or damage to the endothelium. In a Phase II study of endostatin, Heymach and colleagues used four-colour flow cytometry to show that the number of CECs more than doubled in six of seven patients in the first 2 months of therapy (personal communication). Importantly, of the six patients with increased CECs, one had a minor response and four had stable disease for at least 6 months. The patient without an increase developed progressive disease within 6 months. Similar increases in mature CECs have been observed as early as 6 h in four of five patients treated with ZD6126 (Radema *et al*, 2002). The feasibility of measuring markers of early antiangiogenic activity by *ex vivo* analyses of endothelial cells is an encouraging approach. However, further studies are needed to refine the methods and determine whether these and other surrogate markers correlate with clinical outcome.

## CONCLUSIONS

Although angiogenesis inhibitors have great potential as cancer therapeutics, the conventional end points of toxicity and response are inadequate for assessing these agents in clinical studies. The specificity and cytostatic nature of biologic agents requires the development of new methods and surrogate markers to identify doses and schedules with optimal antiangiogenic activity. Understanding the novel mechanisms of angiogenesis inhibitors will facilitate the rational design of surrogate end points.

Although exploratory studies of the use of angiogenic factors in plasma, serum, and urine as surrogate markers have been somewhat disappointing, some tumour types may produce higher levels and more quantifiable factors than other tumours. Thus, angiogenic factors should continue to be studied, especially in more homogeneous patient populations. Further, it is anticipated that the combination of antiangiogenic therapy with traditional cytotoxic agents will offer optimal therapeutic benefit in the management of metastatic disease. Paradoxically, the addition of cytotoxic therapies will introduce toxicities that clinicians hoped to avoid by using angiogenesis inhibitors. Validation of those surrogates suggested to be prognostic indicators of clinical response, for example, urine VEGF, will become critically important for monitoring chemotherapy combinations employing cytotoxic and antiangiogenic drugs. Furthermore, preclinical studies are useful for validating surrogate markers that are hypothesized to be either indirect or direct targets of the investigational agent.

Quantitative analysis of drug–target interactions in tumour biopsies before and after treatment is essential for early proof-of-concept validation, especially during Phase I/II studies. The effects of an angiogenesis inhibitor on signal transduction will probably be evident within hours of exposure to inhibitors of tyrosine kinase receptors. Thus, confirming drug–target effects, that is, the presence of apoptotic endothelial cells, in tumour biopsy specimens obtained early (48 h) after initiation of therapy may provide an assessment of optimal antivascular activity. Indeed, LSC-mediated analysis of apoptosis in tumour cells 48 h after initiation of treatment has proven useful for predicting clinical response to cytotoxic therapies in breast cancer (Davis *et al*, 2003). Nonetheless, numerous surrogate markers for monitoring antiangiogenic activity will probably need to be used in clinical studies to identify an OBD with maximal therapeutic benefit.
